# High-resolution measurements of the multilayer ultra-structure of articular cartilage and their translational potential

**DOI:** 10.1186/ar4506

**Published:** 2014-03-12

**Authors:** Bo He, Jian Ping Wu, Thomas Brett Kirk, John A Carrino, Chuan Xiang, Jiake Xu

**Affiliations:** 1 School of Pathology and Laboratory Medicine, The University of Western Australia, 35 Stirling Highway, Crawley, Western Australia 6009, Australia; 2 Department of Mechanical Engineering, Curtin University, Kent Street, Bentley, Western Australia 6102, Australia; 3 Department of Radiology and Orthopaedic Surgery, Johns Hopkins University, 601 N. Caroline Street, JHOC 5165, Baltimore, MD 21287, USA; 4 The Orthopaedic Department, the Second Hospital, Shanxi Medical University, 56 Xinjian South Road, Yingze, Taiyuan, Shanxi 030001, China; 5 Research Centre for Regenerative Medicine, Department of Orthopaedic Surgery, The First Affiliated Hospital of Guangxi Medical University, 6 Shuangyong Road, Nanning, Guangxi 530021, China

## Abstract

Current musculoskeletal imaging techniques usually target the macro-morphology of articular cartilage or use histological analysis. These techniques are able to reveal advanced osteoarthritic changes in articular cartilage but fail to give detailed information to distinguish early osteoarthritis from healthy cartilage, and this necessitates high-resolution imaging techniques measuring cells and the extracellular matrix within the multilayer structure of articular cartilage. This review provides a comprehensive exploration of the cellular components and extracellular matrix of articular cartilage as well as high-resolution imaging techniques, including magnetic resonance image, electron microscopy, confocal laser scanning microscopy, second harmonic generation microscopy, and laser scanning confocal arthroscopy, in the measurement of multilayer ultra-structures of articular cartilage. This review also provides an overview for micro-structural analysis of the main components of normal or osteoarthritic cartilage and discusses the potential and challenges associated with developing non-invasive high-resolution imaging techniques for both research and clinical diagnosis of early to late osteoarthritis.

## Introduction

The progression of osteoarthritis (OA) has been associated with changes in the morphology and organization of cartilage components [1-3]. Some of these characteristics can be revealed only by high-resolution ultra-structural imaging techniques before biochemical analysis is viable for detecting pathological changes [2]. Compared with biochemical analysis, high-resolution imaging is easy to perform, fast, and straightforward. It allows study of the localization of a specific composite or molecule, the alteration of chondrocyte morphology, and the architectural alteration of fibrillar components in articular cartilage. Most importantly, cartilage imaging combined with various staining reagents will enable us to develop non-invasive tools for the diagnosis and prognosis of OA. This article reviews the multilayered ultra-structure of cartilage components unveiled by high-resolution imaging techniques. The newly confirmed component, elastin, and the potential of high-resolution imaging for OA diagnosis, especially early diagnosis of OA, are also discussed.

## Components of articular cartilage

### Chondrocytes

**Figure 1:**
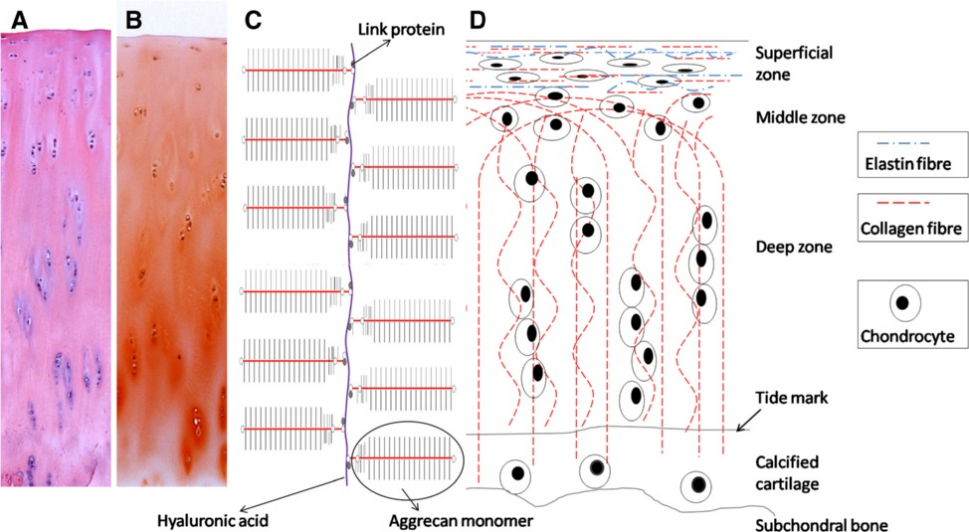
**Zonal organization of articular cartilage (longitudinal view). (A)** Hematoxylin and eosin staining section shows the chondrocytes and extracellular matrix distribution in articular cartilage. **(B)** Safranin O staining section shows that proteoglycan distribution increases from superficial zone to deep zone. **(C)** A schematic image of an aggrecan aggregate. **(D)** The schematic diagram represents the arrangements of collagen and elastin fibers within articular cartilage and changes in chondrocyte shape and arrangement with depth.

Chondrocytes are the resident cells (Figure 1A,D) in articular cartilage, accounting for about 2% of the total volume of articular cartilage [2]. With advanced imaging techniques such as confocal microscopy, researchers have unveiled that each chondrocyte establishes a specialized microenvironment by linking its surface to a transparent pericellular glycocalyx which is confined and enclosed by a fibrillar pericellular capsule [4]. The chondrocyte and its pericellular matrix (PCM) together represent the chondron, which is considered the primary structural, functional, and metabolic unit of articular cartilage [4]. Type VI collagen is the major component of the PCM and exclusively identified within PCM in adult articular cartilage, in spite of the fact that it is widely spread in the extracellular matrix (ECM) of articular cartilage of the newborn [4]. The roles of chondrocytes can be categorized to maintain metabolic balance of articular cartilage under physiological conditions and to mediate mechanotransduction.

### Collagen

Collagens are the most abundant structural macromolecules in the ECM of articular cartilage (Figure 1D) and account for approximately 10% to 20% of the wet weight of articular cartilage [2]. At least 15 distinct types of collagen in articular cartilage are revealed by electron microscopes and confocal microscopes, and type II collagen represents 90% to 95% of the collagens in ECM of articular cartilage [5,6]. Collagen types I, IV, V, VI, IX, and XI are also present but contribute only a minor proportion. These minor collagens help to form and stabilize a fibril network formed by type II collagen [5]. In general, collagen provides articular cartilage with important shear and tensile properties.

### Proteoglycans

Proteoglycans (PGs) represent the second-largest group of macromolecules in the ECM (Figure 1B,C) and account for 10% to 15% of the wet weight of articular cartilage [2,7]. They are large protein-polysaccharide molecules that exist either as monomers or as aggregates [8]. PG monomers consist of a protein core to which about 150 glycosaminoglycan (GAG) oligosaccharides are covalently attached (Figure 1C). These GAG chains extend from the protein core, remaining separated from one another because of charge repulsion. In native cartilage, most PG monomers associate with hyaluronate to form PG aggregates. In these PG aggregates, up to 150 PG monomers non-covalently attach to a central hyaluronic acid by small glycoproteins called link proteins [8]. In articular cartilage, PGs provide hydration and swelling pressure to the tissue, enabling it to withstand compressive forces and deformations [2,7].

### Tissue fluid

Tissue fluid is the most abundant component of articular cartilage and it is mainly water with gases, metabolites, and many free mobile cations (for example, Na^+^, K^+^, and Ca^2+^) which balance the negatively charged PGs in the ECM [9,10]. In general, the fluid component of articular cartilage permits nutrient and waste product movement back and forth between chondrocytes and the surrounding nutrient-rich synovial fluid [11,12]. More importantly, the tissue fluid interacts with collagen and PG in articular cartilage and assists in providing the tissue with its ability to resist compression and return to normal shape after deformation [13].

### Elastin fibers

Elastin fibers are macromolecules comprising a central core of abundant and homogeneous amorphous elastin surrounded by microfibrils such as fibrillin 1 [14]. Elastin fiber in articular cartilage is not well documented as previous histological studies suggested that little elastin fiber was presented in articular cartilage [15]. However, with the microscopic development in confocal microscopy and multi-photon microscopy, researchers have recently unveiled an extensive network of elastin in equine [16], bovine [17,18], and kangaroo [19-21] articular cartilage. These elastin fibers are concentrated mainly in the articular cartilage surface and are aligned parallel to the articular surface. They are closely associated with adjacent collagen fibers and chondrocytes.

The function of elastin fiber in articular cartilage has yet to be explored, but it is expected to play structural, biomechanical, and protective roles for chondrocytes. To comprehend the precise roles of elastin fibers in articular cartilage, studies that are more advanced are needed. These could include research at the gene and molecular levels, in computer modeling as well as in biological imaging. Structural investigation using contemporary microscopy techniques could address the pathological changes of elastin fibers in OA, such as the volume, orientation, and organization. This knowledge could help researchers and orthopedic surgeons understand the etiology and progression of OA. Moreover, with state-of-art imaging facilities, new diagnostic methods may be developed with a focus on elastin fibers.

## Multilayer structure of articular cartilage

Articular cartilage has a multilayered architecture that can be functionally and structurally divided into four zones from the cartilage surface to the subchondral bone: the superficial zone, the middle zone, the deep zone, and the calcified zone (Figure 1) [2].

### The superficial zone

The superficial zone, also named the tangential zone, is the thinnest layer. The superficial zone is composed of flattened chondrocytes with their long axes parallel to the articular surface. The chondrocytes in this zone, characterized histologically by an elongated appearance, preferentially express proteins that have lubricating and protective functions and secrete relatively little PG [22]. The superficial zone has the highest collagen content. The collagen fibrils in this zone are densely packed and have a highly ordered alignment parallel to the articular surface. In addition, elastin fibers recently have been located in the superficial zone of articular cartilage and could have important implications in the normal function of the tissue [17-21].

Functionally, the superficial zone is the articulating surface that provides a smooth gliding surface. Although it has the lowest compressive modulus and could deform approximately 25 times more than the middle zone, the superficial zone is critical to the tensile and shearing resistance of articular cartilage [2]. Its morphology and components also influence the wear and lubrication mechanism of the joint surfaces. An intact superficial surface permits normal operation of the synovial joint at an extremely low coefficient of friction, and any damage to the superficial cartilage zone may lead to rapid wearing of articular cartilage and subsequent cartilage breakdown.

### The middle zone

The middle zone, also termed the transitional zone, has a larger volume than the superficial zone [2]. This zone also possesses a higher compressive modulus than the superficial zone and a more complex collagen structure. The collagen fibrils of the middle zone are packed loosely and aligned obliquely to the articular surface, and the chondrocytes are more rounded than in the superficial layer [2]. It is regarded as a zone in which the structure and components are transitional between the superficial and deep zones.

### The deep zone

The deep zone is also named the radial zone because of its highly radially organized components [2]. The chondrocytes are typically organized in columnar fashion perpendicular to the articular surface. Collagen fibers are large in diameter and oriented parallel to the chondrocyte columns. This layer has the highest PG concentration and thus has the highest compressive modulus [2].

### The calcified zone

The calcified zone separates from the deep zone from the subchondral bone by the tidemark and acts as a transition from soft hyaline cartilage to bone. Chondrocytes in the calcified zone are small and contain almost no endoplasmic reticulum. In some locations, the cells are completely surrounded by calcified ECM and have very little metabolic activity [2].

## High-resolution image techniques

### Spectrum of medical imaging modalities

Current medical imaging methods to look at cartilage and joints include radiography, computed tomography (CT), magnetic resonance imaging (MRI), and scintigraphy (isotope scan). The medical image serves as a surrogate record of morphology and sometimes physiology. A probe, or energy source, is applied to a patient whereby there is a physical interaction that alters the probe changing the energy output and this is recorded by a detector. Some modalities use ionizing radiation, either from electrons (for example, radiography, CT) or from the nucleus (for example, scintigraphy), whereas others use non-ionizing sources, such as radiofrequency (MRI) or sonication (ultrasound, or US). Acquisition may be projectional, such as with the radiography or planar scinitgraphy (where there is overlap of structures in a single projection), or cross-sectional, such as with CT, US, and MRI (where thin slices are created to eliminate the volume-averaging effect of overlapping structures). The pertinent parameters for cartilage imaging are spatial resolution and contrast resolution. Spatial resolution refers to the ability to see spatial detail (that is, resolve two points as different). Radiography has the greatest in-plane spatial resolution (0.1 to 0.2 mm) and as such is well suited for assessing the joint space width (JSW). JSW is an important parameter for clinical trials, particularly of knee OA, and is best evaluated with a weight-bearing examination. Load-bearing imaging is also facilitated by radiography embodiments because they allow standing acquisitions. CT has intermediate spatial resolution (0.2 to 0.3 mm) and is useful to elucidate the bone abnormalities associated with OA that includes marginal osteophytes and the subchondral alterations of sclerosis and cysts. Contrast resolution refers to the ability to distinguish between signal values at different locations and requires some nominal change over the background signal. MRI is advantageous in this regard because of its superb contrast resolution and ability to perform different types of pulse sequences to exploit a variety of soft tissue contrasts not available by other modalities.

### Magnetic resonance image

#### Basic principles

The process of generating a magnetic resonance image depends on the physical properties of the atom related to magnetism and radio waves. Atoms have the property of spin and, when exposed to an external magnetic field, cause the nuclei to process. When a radiofrequency pulse is applied, the atoms are excited, and when it is removed, the nuclei then relax and ‘echo’ back signal. As dissipation occurs, a receiver coil is used to record the signal and computational analysis is performed to create an image. The type of image ‘weighting’ employed during image acquisition determines MRI contrast. The most commonly used images are T1-weighted, T2-weighted, intermediate-weighted (IW) (also known as proton density), and short tau inversion recovery. These types of images may be generated by using various sequence designs. T1 describes relaxation into a condition of thermal equilibrium with the surroundings. T2 describes relaxation of energy traded within the system. The different pulse sequences extract different magnetic properties that infer specific tissue components. On T1-weighted images, fat is bright (unless it has been suppressed, such as in post-contrast studies) and fluid is dark. IW, or proton density-weighted images, are so called because they minimize T2-weighting (by having a short echo time, TE) and minimize T1-weighting (by having a long repetition time) and thus have a contrast that is intermediate to T1 and T2. Fluid appears bright on T2-weighted images and relatively bright on IW images. Fat is variable, depending on whether fat suppression is employed (sometimes done to improve conspicuity). Atoms other than hydrogen (for example, sodium) may be imaged with special multi-nuclear coils.

#### Main applications

A variety of MRI techniques have been developed for the assessment of articular cartilage. These techniques can be broadly used for two different purposes: morphological (structural) assessment and compositional (biochemical) assessment. Whereas morphological assessment predominates in current clinical practice, the increasing use of high-field MRI (3T and above) has enabled the exploration of a number of techniques in compositional cartilage assessment, which will not be described here. MRI spatial resolution is limited by gradient coil performance and the time needed for image acquisition balanced against patient tolerance and motion-induced artefacts. For two-dimensional imaging techniques, the in-plane resolution may approximate 300 μm (0.3 mm), and for three-dimensional acquisitions 400 to 600 μm (0.4 to 0.6 mm), depending on joint size and volume needed to include in field of view. A commonly used pulse sequence for cartilage assessment generates an IW (between T1 and T2 parameters) image which improves cartilage conspicuity, without or with fat suppression (the latter generates a fluid-sensitive sequence for detection of bone marrow lesions).

**Figure 2:**
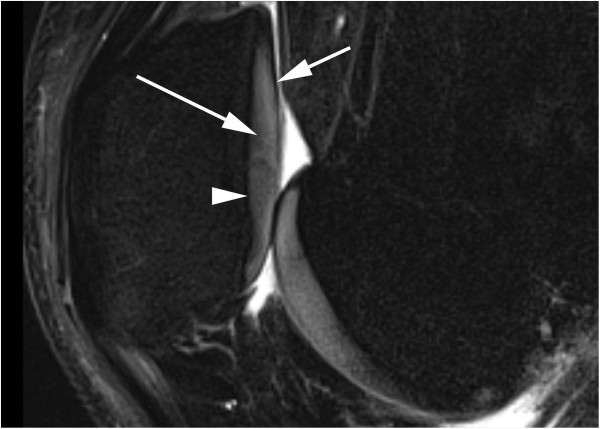
**Magnetic resonance image of normal articular cartilage.** Sagittal fat saturated intermediate-weighted fast spin-echo - repetition time/echo time: 3100/35 - shows normal articular cartilage at the patellofemoral joint with clear demarcation between the articular cartilage and the subchondral bone. Note three-layered cartilage appearance: deeper dark layer (arrowhead), intermediate bright layer (long arrow), and superficial dark layer (short arrow) of lamina splendans.

MRI techniques are capable of both directly and indirectly visualizing cartilage structures. If one accounts for the limitations inherent in MRI, the technique provides a non-invasive means of evaluating the internal structure of the cartilage matrix. Multilayered differentiation is possible in thicker-cartilage areas, such as in patella on the high-resolution and high-field strength imaging (Figure 2). More commonly, articular cartilage has a trilaminar appearance on conventional fluid-sensitive MRI techniques, as a low-signal deep (tidemark and radial zone) layer, a thicker intermediate to bright middle layer (deep zone), and a thin low-signal surface layer (superficial and middle zones). This regional variation in the signal intensity is due largely to T2 value variations caused by the orientation of the collagen fibrils relative to the magnetic field [23]. There is a link between tissue architecture and the MRI image by demonstrating T2 anisotropy within cartilage [24]. When images are acquired with the articular surface perpendicular to the magnetic field (B_0_), the trilaminar appearance is nicely seen with a higher-signal intensity or intermediate transitional layer (thicker middle layer), which separates the low-signal-intensity surface from the low-signal intensity deep layer adjacent to the subchondral bone. Depth-dependent variability in T2 causes a characteristic layered appearance on MRI images of cartilage. These layers reflect the continuous variation in T2 values across the thickness of the tissue [25]. T1 relaxation, proton diffusion, and proton density have only minimal influence on the tissue contrast [26]. The minimum T2 relaxation time in articular cartilage is relatively short, approximately 10 msec. T2 anisotropy is due to the influence of matrix structure on water mobility. As a result, T2 value is the major determinant of the tissue contrast even on T1-weighted and proton density-weighted images [27]. Changes in T2 correlate with changes in the matrix orientation as displayed on fracture-sectioned cartilage on scanning electron microscopy (SEM) [28]. Changes in T2 correlate with changes in polarized light microscopy **(**PLM) [29]. PLM is limited by the use of routine sectioning which cuts through the three-dimensional organization of cartilage. T2 is neither uniform nor constant. It is determined in large part by the orientation relative to B_0_ of both the joint surface and the internal structure of the matrix. This creates predictable challenges to interpreting the significance of T2 measurements. Normal variations in cartilage signal due to underlying bony curvatures and collagen orientation relative to the magnetic field are smooth and gradual, whereas true cartilage abnormalities usually demonstrate abrupt signal change apart from altered morphology. The various cartilage lesions detectable by MRI include chondromalacia, fibrocartilage formation, chondrocalcinosis, fissuring, partial thickness defect, flaps, delamination, and full-thickness cartilage loss.

MRI remains the clinical imaging modality of choice for morphological and compositional evaluation of the articular cartilage [30-32]. Accurate detection and characterization of cartilage lesions are necessary to guide medical and surgical therapy and are critical in longitudinal studies of cartilage. Recent work using 3.0 T MRI systems shows promise in improving the detection and characterization of cartilage lesions, particularly with the advent of robust three-dimensional sequences, which allow detailed morphological assessment of cartilage in arbitrary imaging planes [33-35]. Development of MRI systems with ultra-high field strength, multi-channel coils, and more signal-to-noise ratio-efficient sequences may allow the implementation of biochemical sequences in clinically feasible scan times to allow early detection of cartilage lesions before they become morphologically apparent. As coil technology further improves and pulse sequences become more sophisticated, high-resolution and high-contrast morphological and biochemical assessment of cartilage will likely play an important role in the management of patients with articular cartilage injuries and degeneration.

### Compositional (biochemical) imaging of cartilage

#### Basic principles

Morphological changes in cartilage are accompanied by biochemical changes in the ECM that reflect the altered biochemical properties of the tissue. These biochemical changes can be targets for a host of advanced MRI techniques capitalizing on a variety of ‘compositional’ alterations in the extracellular milieu.

##### T2 mapping

**Figure 3:**
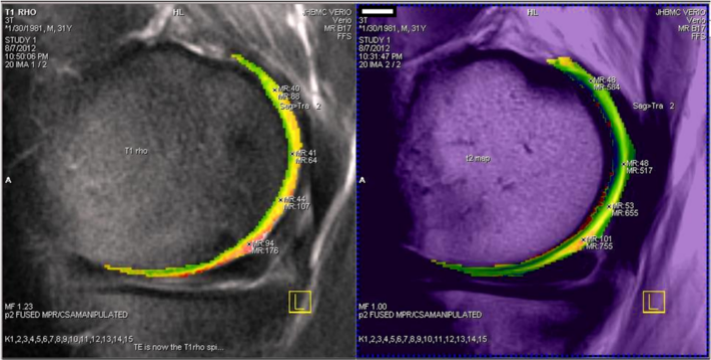
**Magnetic resonance imaging and biochemical imaging of normal articular cartilage.** Sagittal intermediate-weighted fast spin-echo - repetition time/echo time: 3100/35 - with superimposed biochemical imaging colorized maps (left = T1 rho and right = T2) shows normal articular cartilage. Note three-layered cartilage appearances.

T2 mapping measures the collagen content within the ECM of cartilage by assessing the changing interactions between water molecules and collagen. T2 maps are strongly influenced by orientation of collagen molecules and dipole-dipole interaction anisotropy [36]. Thus, T2 mapping values increase from deep to superficial layers in the healthy articular cartilage (Figure 3, right). Any injury to the cartilage in the form of degeneration or trauma (or both) increases the amount of internal free water and therefore increases the T2-signal intensity [37]. Decreased T2 values reflect water-content loss seen in cartilage degeneration, fibrocartilage, or chondrocalcinosis. T2 maps have been found to correlate with activity levels in asymptomatic subjects [38].

##### Ultra-short echo time imaging

Conventional clinical scanners typically employ higher TEs (≥10 msec) for routine T2-weighted sequences. MRI signals from musculoskeletal tissues with short T2 characteristics, such as deep radial and calcified layers of cartilage, decay rapidly and therefore produce little or no signal on conventional sequences. With ultra-short TE MRI, signal from tissues with predominantly short T2 (and T2*) can be detected by using TEs that are 10 to 1,000 times shorter than those used in conventional imaging sequences, enabling visualization of layers that normally are not depicted [39]. The region of the osteochondral junction consisting of the calcified cartilage layer and subchondral bone is important for solute transport between the vasculature and articular cartilage and has been implicated in the pathogenesis of OA. Changes beginning in the calcified cartilage layer may then propagate superficially and result in further cartilage degeneration [40].

##### Diffusion-weighted imaging

Measuring the spatial restriction of diffusivity (in contrast to unrestricted diffusion in free water) according to the tissue’s ultra-structure gives information about the tissue’s structural properties. Diffusion measurements are obtained by applying multiple diffusion-sensitizing gradient MRI pulses to generate magnetization in water molecules. When diffusion-sensitizing gradients are applied, unrestricted water gains a random amount of phase and does not refocus, which results in signal loss of the tissue undergoing diffusion [41]. The diffusion of water in articular cartilage reflects the biochemical structure and architecture of the tissue. The apparent diffusion coefficient (ADC) is low in healthy cartilage because the diffusion of water molecules is restricted by collagen anisotropy. Disruption of the cartilage matrix results in enhanced water mobility, which increases the ADC of cartilage [42].

##### Sodium magnetic resonance imaging

Any atom with an odd number of protons or neutrons (or both) possesses a nuclear spin momentum that can be exploited for MRI. Sodium, such a positive ion, is found in higher concentration in the interstitium rather than in synovial fluid secondary to its attraction to anionic GAG charge. Therefore, a higher concentration of sodium is found in areas with high GAG concentration [43]. Sodium atoms are the main component of high fixed charge density present in the PG sulfate and carboxylate group. Cartilage degeneration leads to loss of PG content and hence loss of GAG and fixed charge density, resulting in loss of sodium ions from the tissue [44]. Sodium imaging has been shown to be sensitive to small changes in PG concentration in studies using *in vivo* high-field *in vitro* cartilage specimens [35].

##### T1 rho imaging

T1 rho (T1ρ) technique has been used to assess low-frequency interactions between hydrogen and macromolecules in free water. Capturing several values allows one to solve for the slope of the decay function and create either gray-scale or color-coded maps depicting T1 mapping without the need for an intra-articular paramagnetic agent [45]. Damaged hyaline cartilage demonstrates higher T1ρ values than the normal cartilage, and T1ρ imaging has higher sensitivity than T2-weighted imaging for differentiating between normal cartilage and early cartilage degeneration in OA [46]. T1ρ values also showed regional variations within cartilage, with the highest values in the superficial zone, decreasing in the middle zone, and increasing near the subchondral bone (Figure 3).

##### Delayed gadolinium-enhanced magnetic resonance imaging of cartilage

When anionic molecules such as those of gadopentetate dimeglumine (Gd-DTPA^2-^) (negatively charged) enter the cartilage, they concentrate in areas where GAG content is relatively low. After the intravenous administration of Gd-DTPA^2-^, a quantitative assessment of GAG content can be obtained by performing T1 mapping of the cartilage. The term ‘delayed’ in delayed gadolinium-enhanced magnetic resonance imaging of cartilage (dGEMRIC) reflects the time needed to allow articular tissue penetration of Gd-DTPA2 [47]. Typically, the joint is exercised for 10 to 20 minutes after Gd-DTPA^2-^ is administered, following which T1 measurements are performed at approximately 60 minutes after injection. A T1 map of the cartilage allows assessment of the GAG content, with lower values corresponding to areas of GAG depletion. The T1 measurement after penetration of Gd-DTPA^2-^ is referred to as the dGEMRIC index. Areas of cartilage with a lower dGEMRIC index are observed in cases of OA [48].

##### Glycosoaminoglycan concentration by chemical exchange-dependent saturation transfer imaging

Glycosoaminoglycan concentration by chemical exchange-dependent saturation transfer (gagCEST) is a technique based on labile protons residing on the GAGs. Both amide proton and hydroxyl protons from GAG have been shown to be suitable as chemical exchange-dependent saturation transfer agents, allowing the direct measurement of GAG content *in vivo*[49]. A high correlation exists between gagCEST and sodium MRI [50].

### Electron microscopy

#### Basic principles

SEM and transmission electron microscopy (TEM) are two types of electron microscopy used to image the ultra-structure of articular cartilage. Electron microscopes use accelerated electrons instead of visible lights for imaging. In SEM, the electrons interact with electrons in the sample, producing various signals that contain information about the sample’s surface topography and components. By collecting the scattered electrons, an image of the surface structure of the specimen is produced. In TEM, a beam of electrons is transmitted through an ultra-thin specimen and interacts with the specimen as it passes through. An image is formed from the interaction of the transmitted electrons and only the electrons passing through the sample are illuminated in the image.

#### Main applications

So far, electron microscopy has been used to reveal many facets of the physiology and pathology of articular cartilage. An SEM study on the collagen organization of articular cartilage from young to adult specimens has suggested that structural remodeling of the collagen network occurs in the first months after birth [51]. Through the remodeling, the collagen network is transformed from a uniform structure at birth to a typical arcade-like ‘Benninghoff structure’ in adults. SEM has also provided details of the collagen organization in normal, degenerated, and repaired human articular cartilage [52]. This detailed assessment of collagen architecture could benefit the development of cartilage repair strategies intended to recreate functional collagen architecture. Similarly, electron microscopes have been applied to study the morphological and physiological changes in chondrocytes. A recent TEM study has disclosed a diminished capacity of aged bovine chondrocytes to produce a competent ECM with respect to collagen volume and mechanical functions [53].

In addition to the studies of chondrocytes and collagen fibrils, electron microscopes have been applied in visualizing the ultra-structure of PGs in articular cartilage. SEM imaging enables researchers to distinguish two structurally distinctive populations of PG aggregates [54]. The differences between the two types of aggregate, in particular the number of aggrecan molecules per aggregate, may reflect differences in their assembly, stability, and turnover, which therefore give them different mechanical and biological properties [54].

#### Advantages and limitations

Electron microscopes have been proven to be an invaluable tool for examining the ultra-structure of articular cartilage under healthy, diseased, and repair conditions (Table 1). Apart from imaging cellular and fibrillar components of articular cartilage, the most remarkable characteristic of electron microscopes is the capability of imaging PG which could not be resolved by optical microscopes (Table 1). This makes it possible for researchers to assess diseased or engineered cartilage and appraise its compressive property by investigating the morphology of PG, such as density, length, and the magnitude of aggregation. It is the high magnification and superior imaging resolution capability that makes electron microscopes surpass any available light microscopes in providing detailed structural information of articular cartilage (Table 1). However, researchers should be aware of the limitations of this technique. For example, electron microscopes are not applicable to fresh tissue or live cell imaging, and specimen preparation can be technically very complex. A sample prepared for SEM imaging requires fixation, coating, and dehydration for electron conduction. These processes are inevitably associated with changes of cartilage characteristics, leading to the potential for images to contain various degrees of artefact and may not reflect the real architecture of articular cartilage *in vivo*. For instance, cartilage surface phenomena such as undulations, pits, and humps have been suggested to be shrinkage artefacts due to dehydration during cartilage processing for SEM but also may be structural characteristics [55].

**Table 1:** Comparative features and guidelines for applying contemporary imaging techniques in imaging components of articular cartilage

**Imaging techniques**	**Comparative features**								
	**Chondrocyte**	**Collagen**	**Elastin**	**Proteoglycan**	**Resolution**	**Depth**	**Cost**	**Time-consuming**	**Friendly access**
MRI					+		++	+	++
Electron microscopy	+	++		+++	+++		+++	+++	+
CLSM	+++	+++	+++		++	++	++	+	+++
SHG	+	+++			++	+++	++	+	++
LSCA	++	+			++	++	+	+	+++
SHG arthroscope (potential)	+	+++			++	+++	++	+	+++

+ average, ++ recommended, +++ highly recommended. CLSM, confocal laser scanning microscopy; LSCA, laser scanning confocal arthroscopy; MRI, magnetic resonance imaging; SHG, second-harmonic generation.

### Confocal laser scanning microscopy

#### Basic principles

Confocal laser scanning microscopy (CLSM) is one of the most commonly used imaging techniques in biological and medical science. It differs from fluorescence microscopy in two major factors: light source and a pinhole. The light source in a CLSM is usually a laser which produces monochromatic light. Compared with mercury vapor lamp or light-emitting diodes in fluorescence microscopy, a laser possesses unique properties for being an ideal illumination source in CLSM, including a high degree of monochromaticity, small divergence angle, high brightness, and a high degree of spatial and temporal coherence [56]. Another characteristic of CLSM is the use of a pinhole. A confocal pinhole (aperture) is placed at a very precise position in the optical path such that emitted lights coming from the plane of focus will pass through it and enter the photon detector. The out-of-focus emission light will be blocked outside of the pinhole. Thus, the image quality is significantly improved. Point illumination and point detection due to the pinhole result in improved resolution and contrast in images compared with those seen in wide-field microscopes.

#### Main applications

**Figure 4:**
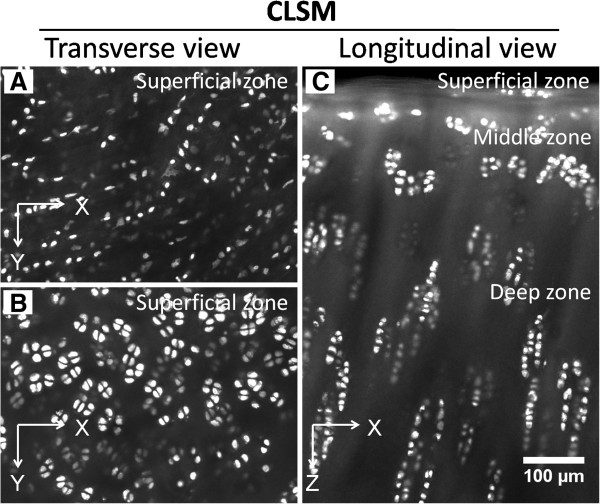
**Confocal laser scanning microscopy (CLSM) images of chondrocytes in articular cartilage. (A,B)** Transverse view of arrangements of chondrocytes (strings and pairs) in the superficial zone of articular cartilage of different loading locations. **(C)** Longitudinal view of articular cartilage from superficial zone to deep zone. Images were taken from an acriflavine-stained fresh femoral condyle articular cartilage sample from kangaroo. X and Y coordinates indicate images that were parallel to the cartilage surface and taken from a transverse view; X and Z coordinates indicate images that were vertical to cartilage surface and taken from a longitudinal view.

**Figure 5:**
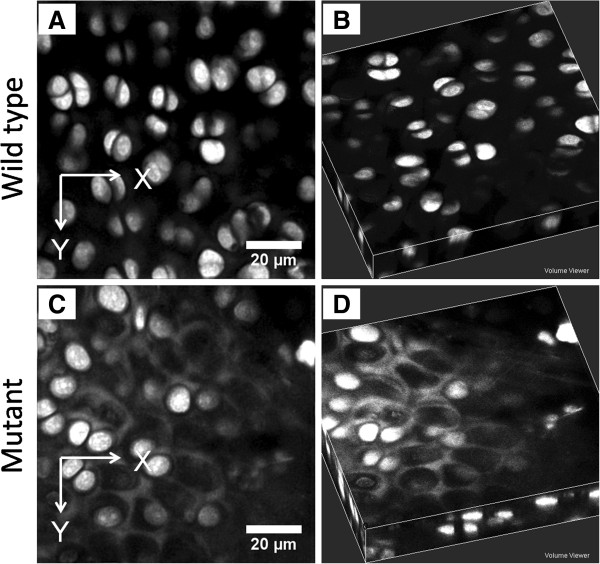
**Confocal laser scanning microscopy images showing cellular differences between wild-type and dedicator of cytokinesis 2 (*Dock2*) gene mutant mice. (A,B)** The morphology of healthy chondrocytes in the superficial zone of articular cartilage from wild-type mice. **(C,D)** The loss of chondrocytes in the superficial zone of the articular cartilage from *Dock2* gene mutant mice. X and Y coordinates indicate images that were parallel to the cartilage surface and taken from a transverse view.

In the field of cartilage research, CLSM is widely used in the study of chondrocytes. Figure 4 shows typical images of chondrocytes in native kangaroo articular cartilage. The organization of chondrocytes from different loading locations (Figure 4A,B) and the zonal arrangements of the chondrocytes from cartilage surface to the deep zone have been clearly demonstrated (Figure 4C). CLSM has also shown its capacity to detect physiological changes in chondrocytes with a specific gene mutation (Figure 5). Dedicator of cytokinesis 2 (*Dock2*) is a protein involved in intracellular signaling networks and closely related to lymphocyte migration and maturation [57]. Using *N*-ethyl-*N*-nitrosourea to induce single-nucleotide mutation of *Dock2* gene [58], a CLSM study has revealed the loss of chondrocytes in the superficial zone of femoral condyle articular cartilage of mice (Figure 5). CLSM has also assisted in unraveling chondron structure [4], changes of chondrocytes in cartilage pathology [1,3], factors affecting chondrocyte physiology [59], and the role of chondrocytes in ECM metabolism [60]. Because of the pivotal role of chondrocytes in articular cartilage, the utilization of CLSM in chondrocyte imaging will surely continue to benefit the study of articular cartilage.

**Figure 6:**
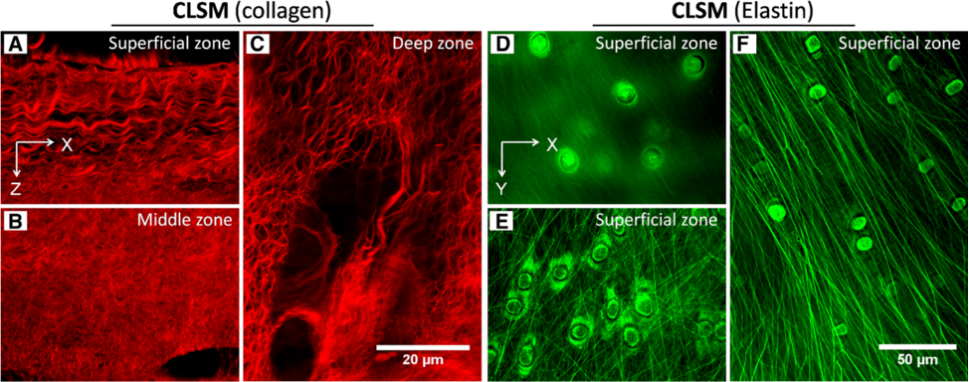
**Confocal laser scanning microscopy (CLSM) images of collagen and elastin in articular cartilage. (A-C)** Images showing zonal organization of collagen fibers were taken from picrosirus red-stained slides of articular cartilage from kangaroo femoral condyle. These images resemble Figure 2 of our article in [19]. **(D-F)** Images showing different organizations of elastin fibers in the superficial zone of articular cartilage of kangaroo femoral condyle. These images resemble Figure 7 of our article [19]. X and Y coordinates indicate images that were parallel to the cartilage surface and taken from a transverse view; X and Z coordinates indicate images that were vertical to cartilage surface and taken from a longitudinal view.

CLSM is also a valuable tool for studying the ECM components of articular cartilage [61]. With picrosirius red staining, CLSM has led to disclosure of the multilayer structure of collagens in kangaroo articular cartilage (Figure 6A-C). The high-resolution confocal images clearly depict the heterogeneous arrangements of collagen fibrils in different zones and their relationship with chondrocytes. Through the application of elastin-specific fluorescent dye, sulforhodamine B (SRB), the localization and organization of elastin could also be studied. Although elastin fibers are suggested to be absent in articular cartilage by conventional histological techniques [19], CLSM study has revealed a significant presence of elastin fibers in kangaroo cartilage surface and around the chondrocytes (Figure 6D-F). These data concerning the orientation and organization of the elastin fibril structure in articular cartilage will help to establish numerical models that assist in understanding cartilage physiology and pathology. With appropriate fluorescent dyes, the role of CLSM for orthopedic research and clinical application is far-reaching.

#### Advantages and limitations

The widespread use of CLSM for articular cartilage biology is due to its impressive merits (Table 1). In CLSM, only one single point in microscopic objects is illuminated at any time and this point is then imaged into the pinhole at the entrance of the photo-detector while out-of-focus signals are blocked. The ability to acquire images with improved axial resolution using CLSM is especially valuable for imaging thick specimens with complex fluorescent labeling distributed over a broad depth. CLSM also allows optical sectioning of tissues, and three-dimensional reconstruction can be built from image stacks, enabling the localizing of components in articular cartilage and the study of interaction between several macromolecules. Compared with electron microscopy (Table 1), CLSM is less expensive and less time-consuming and allows *in situ* investigation of the composites of articular cartilage without processing of the samples.

Despite the above advantages, CLSM has its limitations. The high-resolution image from CLSM comes at a price of reduced speed as compared with wide-field microscopy. It takes more time to scan an image using CLSM than using conventional optical microscopic methods, and the image size is usually much larger. Furthermore, the high-intensity laser excitation used in the CLSM system can cause photo damage in some circumstances, and thus an optimum laser power setting is important for imaging.

### Second-harmonic generation microscopy

#### Basic principles

Second-harmonic generation (SHG) microscopy, which is based on the SHG non-linear optical effect, has emerged as a powerful optical imaging modality. In the SHG process, photons interact with a non-linear material and form new photons with twice the energy of the initial photons. Generation of SHG signals requires a short-pulse laser which gives very high light intensities, and the frequency-doubled SHG signal can be separated from excitation light (usually a wide range of wavelengths in the infrared region) by a set of appropriate filters. SHG microscopy allows a very high axial and lateral resolution comparable to that of CLSM without the use of pinholes.

#### Main applications

In biological and medical science, SHG microscopy is often used for imaging collagen. Collagen fibrils, which have a triple-helical structure and are both non-centrosymmetric and highly crystalline, are by far the strongest source of second harmonics in animal tissue. Given the significant functions of collagen in articular cartilage, SHG microscopy could have profound implications for cartilage studies.

**Figure 7:**
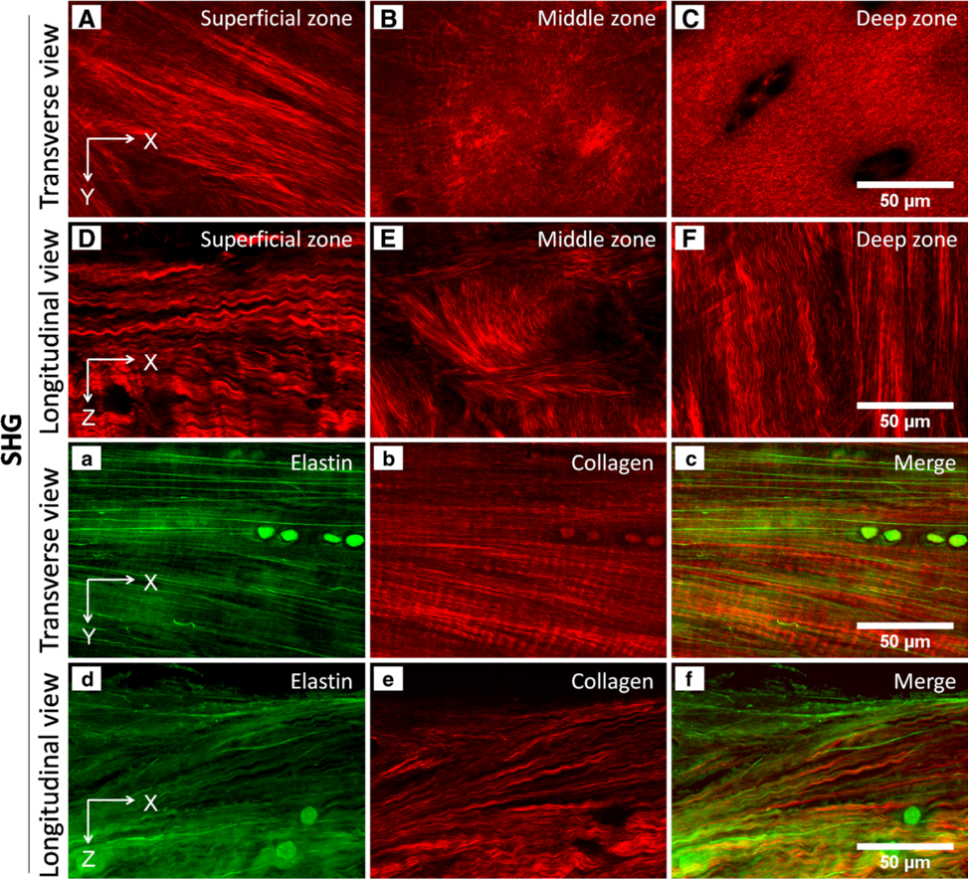
**Second-harmonic generation (SHG) images of collagen and the relationship between collagen and elastin fibers. (A-F)** SHG images of collagen which show its zonal structure were taken from fresh articular cartilage samples from kangaroo femoral condyle from both transverse and longitudinal views. These images resemble Figure 3 of our article [19]. **(a-f)** Collagen and elastin fibers in the superficial zone of articular cartilage from both transverse and longitudinal views and their relationships. Collagen was imaged by collecting SHG signals, and elastin fires were imaged by collecting sulforhodamine B fluorescent signals. Images **(a-f)** resemble Figures 5 and 6 of our article [19]. X and Y coordinates indicate images that were parallel to the cartilage surface and taken from a transverse view; X and Z coordinates indicate images that were vertical to cartilage surface and taken from a longitudinal view.

SHG microscopy possesses several characteristics enabling it be an ideal tool for studying articular cartilage (Table 1). First, SHG microscopy provides detailed information of collagen *in situ* and enables the study of multilayer structure of articular cartilage. As shown in Figure 7 (A-F), high-resolution SHG images show that the heterogeneous collagen is organized in the superficial, middle, and deep zones of fresh kangaroo articular cartilage in both transverse and longitudinal planes. Changes in the orientation and organization of collagen from the superficial to the deep zone of articular cartilage can be clearly depicted by the SHG images.Second, in combination with fluorescent dyes, SHG microscopy can be used to image several components in articular cartilage simultaneously, thus enabling an understanding of the relationship between collagen and other ECM components. Figure 7 (a-f) shows the plausibility of simultaneous imaging of collagen from its SHG signal and elastin fiber from SRB fluorescence from fresh kangaroo articular cartilage. From these images, we argue that the orientation of the elastin fibers is in general parallel to that of collagen in the superficial zone of articular cartilage. This is achievable because the SHG signal is both strictly defined in wavelength and propagated strongly forward, which does not interfere with the fluorescence of a fluorescent probe (for example, the SRB fluorescence in Figure 7a-f). As a result, the SHG signal can be distinguished from the fluorescence, and, in essence, the collagen can be imaged independently when even multiple fluorescent probes are used.

#### Advantages and limitations

There are several advantages to using SHG microscopy to study collagens. SHG microscopy is superior to electron microscopy in terms of damage to the articular cartilage and allows an even larger depth of imaging than CSLM (Table 1). SHG signals arise from an induced polarization rather than from absorption, which leads to substantially reduced photobleaching and phototoxicity relative to electron or fluorescence methods [62]. The use of lasers at the near-infrared spectral range enables SHG microscopy to achieve high-resolution imaging to depths of several hundred microns [62]. With future improvement in laser intensity, SHG microscopy will be a vital tool for living tissue imaging.

However, when using SHG microscopy for cartilage imaging, researchers should take several issues into consideration. SHG microscopy uses lasers at infrared wavelengths for excitation. However, before the detector, almost all the infrared-blocking filters used to block the excitation beam do not allow any wavelength shorter than 400 nm through**.** Therefore, when SHG microscopy is used, it is suggested that the excitation laser be tuned to 830 nm or longer (to allow the emission wavelengths longer than 400 nm) and that the characteristics of the blocking filter be checked.

### Confocal arthroscopy

#### Basic principles

Laser scanning confocal arthroscopy (LSCA) incorporates high-magnification and high-resolution confocal technology within the confines of an arthroscopic probe to provide the imaging requirements necessary to perform detailed assessment of the condition of the cartilage. It employs an imaging principle similar to that of CLSM and differs from CLSM with a unique miniaturized laser scanning mechanism. In LSCA, a focused laser light source illuminates a small volume within the specimen. The incident laser light excites the contrasting agent in the tissue, and the excitation wavelengths travel along the same optical path before particular wavelengths are filtered by a longpass filter into a photodetector. Out-of-focus light rejection in the LSCA is achieved by use of the launch fiber end as the detector confocal aperture. By raster scanning of the single excitation point, a fully in-focus image is thus created. A previous LSCA provided a spatial resolution of 2 μm, with an image refresh rate of 2 Hz [63]. A more recent confocal arthroscope offers a lateral imaging resolution of 0.7 μm and an axial imaging resolution of 7 μm [64].

#### Main applications

In the field of orthopedics, the LSCA has been used as an optical biopsy tool and an *in vivo* imaging tool in articular cartilage, bone, ligament, muscle, menisci, and synovium [65]. Its application in both OA cartilage study and cartilage repair assessment has also been explored [66]. LSCA has permitted diagnosis of classic OA changes such as clustering of chondrocytes, fissures, and surface fibrillations in the articular cartilage of femoral condyle, tibial plateau, and patella. Correlation analysis demonstrated significant correlations between histological assessments and LSCA modality. The capability of the LSCA technique for characterizing OA features has been further confirmed by using human tibial plateaus acquired from total knee arthroplasty surgeries [63]. The efficacy of LSCA was also proved in an ovine model of articular cartilage injury repair [67]. LSCA confocal images can reveal clear differences between native cartilage, matrix-induced autologous chondrocyte implantation repair cartilage, fibrous cartilage, and degenerative tissue.

#### Advantages and limitations

LSCA has several advantages for cartilage imaging and evaluating OA progression (Table 1). First, this technique is viable for non-destructive imaging of the micro-structure of OA cartilage and has the benefits of elucidating the exact pathology of the initial stages of OA. To assess the micro-structural changes in OA, especially early OA, conventional methods use histological analysis which requires taking biopsy samples from patients. The biopsy process itself is inherently destructive to the tissue, and subsequent cartilage pathology may be induced as the self-repair ability of articular cartilage is highly limited. Thus, a non-destructive examination technique is necessary and would help orthopedic clinicians choose appropriate repair strategies. The emergence of LSCA has circumvented the drawbacks brought by biopsy and has demonstrated its abilities for non-destructive imaging of the micro-structure of both OA cartilage [63,66] and repaired cartilage [67]. LSCA also provides an easy, efficient, and less labor-intensive method to study the changes in articular cartilage as compared with histological techniques.

Despite the demonstrated advantages of LSCA technology as a non-destructive assessment tool for cartilage micro-structure, there are several concerns about its usage in orthopedic clinics. An appropriate contrasting agent should be chosen with the greater penetration to the cartilage tissue as it determines the penetration of LSCA. Another issue is the potential hazard introduced by using a contrasting agent. Prior to *in vivo* imaging in human subjects, the potential toxicity of fluorophores to chondrocytes and articular cartilage must be investigated. Fluorescein is currently used for LSCA and is approved for direct systemic application in human subjects. However, acridine orange, a commonly used fluorescent contrast agent in animal studies, can differentiate specific DNAs and RNAs in nuclei and cytoplasm but has distinct cellular effects, including inhibition of mitosis and the induction of bi-nucleation in chondrocytes [68]. It has also been noted that the combination of intense laser light illumination and potential fluorophore toxicity may damage living cells. Hence, the safety of the LSCA technique must be confirmed before any widespread clinical use can be advocated, and this remains the focus of ongoing studies.

### Development of non-invasive imaging techniques

#### Potentials of developing second-harmonic generation arthroscopy

Owing to the outstanding capabilities of SHG microscopy discussed above and the plausibility of using LSCA to study the musculoskeletal system, the development of an SHG arthroscopy as a non-invasive imaging technique would be very promising for OA diagnosis. During the early stage of OA, collagen content is initially maintained, but collagen organization is severely perturbed [2]. Therefore, a potential SHG arthroscope would be enormously beneficial to early diagnosis of OA by detecting alteration of SHG signals of collagen organization before the biochemical analysis is viable. Moreover, SHG not only is designed for organizational imaging of collagen in articular cartilage but also is capable of distinguishing different types of collagen. Studies have shown that SHG microscopy discriminates between type I and type III collagen [69] and between type I and type II collagen [70] through a polarization setting. Because the transformation from type II to type I collagen is a significant sign of OA [2], an SHG arthroscope will undoubtedly assist in diagnosis of OA by targeting the transformation between collagen types. In addition, SHG arthroscopy would surpass other imaging techniques, such as electron microscopy, CLSM, and LSCA, for collagen studies as it would involve no destructive tissue biopsy and less phototoxicity to articular cartilage and allow imaging of the tissue with greater depth (Table 1).

#### Challenges for developing second-harmonic generation arthroscopy

One of the biggest challenges for developing an SHG arthroscopy is to minimize the SHG microscope. The potential SHG arthroscopy applies the same physics principle in SHG microscopy to generate SHG signals from collagens, but it should be mobile and have a small and long and thin probe instead of a set of objectives. In SHG arthroscopy, the apparatus to generate a long-wavelength laser needs to be confined to a small size, which could challenge the effectiveness of the generation of the laser and the cooling system and so on. Another concern is the effectiveness of collecting the SHG signal as both the excitation and emission wavelength travel along the same optical path in current LSCA [65,67]. Strategies such as using different sets of filters or introducing two separate fibers for excitation and emission lights should be adopted to test the outcome of the SHG signal. It is also worth introducing several magnification choices for researchers and clinicians to obtain several levels of cellular or subcellular information [65-67].

## Conclusions

Articular cartilage is a tissue with heterogeneous chondrocyte and ECM organization. The components and organization of articular cartilage are critical to its biomechanical properties and their disruption has been associated with cartilage degeneration. Given their significance, the cellular components and ECM as well as the multilayer structure of articular cartilage have been summarized. As new evidence of elastin fibers existing in articular cartilage is emerging, this review further discussed the likely biological and biomechanical functions of elastin as well as its possible role in the diagnosis of OA. In-depth knowledge of elastin fibers in articular cartilage will increase our understanding of the physiology of articular cartilage and benefit in revealing cartilage pathology and in developing engineered cartilage for repair. To achieve these goals, high-resolution imaging techniques definitely have an important role to play. The review also highlighted recent advances in high-resolution imaging techniques and their applications in studying almost all aspects of articular cartilage. Some of these techniques hold promise to unveil the etiology of cartilage degeneration and diagnose early OA. Undoubtedly, the quest for greater knowledge of the high-resolution imaging techniques along with a comprehensive background of articular cartilage biology will help explore the physiology and pathology of articular cartilage as well as develop non-invasive diagnostic tools and efficient repair strategies for OA.

## Abbreviations

ADC: Apparent diffusion coefficient; CLSM: Confocal laser scanning microscopy; CT: Computed tomography; dGEMRIC: Delayed gadolinium-enhanced magnetic resonance imaging of cartilage; *Dock2*: Dedicator of cytokinesis 2; ECM: Extracellular matrix; GAG: Glycosaminoglycan; gagCEST: Glycosoaminoglycan concentration by chemical exchange-dependent saturation transfer; Gd-DTPA^2-^: Gadopentetate dimeglumine; IW: Intermediate-weighted; JSW: Joint space width; LSCA: Laser scanning confocal arthroscopy; MRI: Magnetic resonance imaging; OA: Osteoarthritis; PCM: Pericellular matrix; PG: Proteoglycan; PLM: Polarized light microscopy; SEM: Scanning electron microscopy; SHG: Second-harmonic generation; SRB: Sulforhodamine B; TE: Echo time; TEM: Transmission electron microscopy; T1ρ: T1 rho; US: Ultrasound.

## Competing interests

The authors declare that they have no competing interests.
